# Quality of care in the free maternal healthcare era in sub-Saharan Africa: a scoping review of providers’ and managers’ perceptions

**DOI:** 10.1186/s12884-021-03701-z

**Published:** 2021-03-19

**Authors:** Monica Ansu-Mensah, Frederick Inkum Danquah, Vitalis Bawontuo, Peter Ansu-Mensah, Tahiru Mohammed, Roseline H. Udoh, Desmond Kuupiel

**Affiliations:** 1 Department of Public Health, Faculty of Health and Allied Sciences, Catholic University College of Ghana, Fiapre, Sunyani, Ghana; 2 The University Clinic, Sunyani Technical University, Sunyani, Ghana; 3 Department of Global Health, Centre for Evidence-based Health Care, Division of Epidemiology and Biostatistics, Faculty of Medicine and Health Sciences, Stellenbosch University, Tygerberg, Cape Town, 7530 South Africa; 4 Department of Secretaryship and Management Studies, Sunyani Technical University, Sunyani, Ghana; 5 Research for Sustainable Development Consult, Sunyani, Ghana

**Keywords:** Maternal healthcare, Free healthcare policy, Health financing, Health managers’, Healthcare providers’, Quality of care, Sub-Saharan Africa

## Abstract

**Background:**

Free maternal healthcare financing schemes play an essential role in the quality of services rendered to clients during antenatal care in sub-Saharan Africa (SSA). However, healthcare managers’ and providers’ perceptions of the healthcare financing scheme may influence the quality of care. This **s**coping review mapped evidence on managers’ and providers’ perspectives of free maternal healthcare and the quality of care in SSA.

**Methods:**

We used Askey and O’Malley’s framework as a guide to conduct this review. To address the research question, we searched PubMed, CINAHL through EBSCOhost, ScienceDirect, Web of Science, and Google Scholar with no date limitation to May 2019 using keywords, Boolean terms, and Medical Subject Heading terms to retrieve relevant articles. Both abstract and full articles screening were conducted independently by two reviewers using the inclusion and exclusion criteria as a guide. All significant data were extracted, organized into themes, and a summary of the findings reported narratively.

**Results:**

In all, 15 out of 390 articles met the inclusion criteria. These 15 studies were conducted in nine countries. That is, Ghana (4), Kenya (3), and Nigeria (2), Burkina Faso (1), Burundi (1), Niger (1), Sierra Leone (1), Tanzania (1), and Uganda (1). Of the 15 included studies, 14 reported poor quality of maternal healthcare from managers’ and providers’ perspectives. Factors contributing to the perception of poor maternal healthcare included: late reimbursement of funds, heavy workload of providers, lack of essential drugs and stock-out of medical supplies, lack of policy definition, out-of-pocket payment, and inequitable distribution of staff.

**Conclusion:**

This study established evidence of existing literature on the quality of care based on healthcare providers’ and managers’ perspectives though very limited. This study indicates healthcare providers and managers perceive the quality of maternal healthcare under the free financing policy as poor. Nonetheless, the free maternal care policy is very much needed towards achieving universal health, and all efforts to sustain and improve the quality of care under it must be encouraged. Therefore, more research is needed to better understand the impact of their perceived poor quality of care on maternal health outcomes.

**Supplementary Information:**

The online version contains supplementary material available at 10.1186/s12884-021-03701-z.

## Background

Every country around the globe considers maternal healthcare as one of the top-most importance [1]. It is for this reason that maternal healthcare financing is given much attention in various forms [2]. Member countries in the World Health Organization (WHO) Africa Regions proposed in the 2008 Ouagadougou declaration to achieve better health for all [3] but maternal healthcare is paramount worldwide [4]. In sub-Saharan Africa (SSA), some countries introduced free maternal healthcare financing policies to curb the pressing complications and challenges associated with maternal healthcare delivery in order to achieve the Millennium Development Goal five (MDG 5) [5]. The United Nations Sustainable Development Goal (SDG) 3.1 (reducing maternal mortality to less than 70 per 100,000 live births) aims to build on the MDG 5 achievement [6–8].

Free maternal healthcare financing includes any health financing policy which eliminates all or part of catastrophic healthcare cost for poor pregnant women [9]. It could be in a form of health insurance where a required premium is taken from all subscribers and a prescribed package of service is rendered to each member in the scheme [10]. Sometimes, Lower and Middle-Income countries (LMICs) rely on loans and grants, taxes, and donor support to finance healthcare with major consideration for the vulnerable (women in their reproductive age and children) [11]. Bridging the financial barriers does not only mean removing the maternal healthcare burdens but also ensuring the quality of service delivery and equitable distribution of resources [7]. Quality refers to the standard or expectation that a product or service is required to meet the level of satisfaction for a person or group [4, 11]. Therefore, quality is said to be subjective.

Nonetheless, healthcare managers’ and providers’ perceptions of free maternal healthcare policies may influence the quality of care rendered to beneficiaries [12]. Since research has shown that adequacy and availability of funds, provision of essential drugs and supply, and available human resources facilitate health service quality [13, 14]. Evidence is abundant on how the implementation of free maternal healthcare financing schemes has improved access to maternal health services and outcomes. Nonetheless, there are several reported challenges including a delayed refund of monies to the health facilities in some SSA countries which potentially can affect the quality of care. Despite this, no study has systematically mapped literature for policy decisions or identified literature gaps for future research. Our earlier review focused on women’s perception of the quality of care in the free maternal healthcare era [15]. Therefore, this current scoping review mapped evidence on providers’ and managers’ perceptions of the quality of care in the free maternal healthcare era in SSA.

## Methods

We used Arksey and O’Malley’s framework as a guide to conduct a scoping review. The protocol of this study was developed and published elsewhere [16]. We followed the preferred reporting items for systematic reviews and meta-analyses extension for scoping reviews checklist to report this study.

### Identifying the research questions

The research question for this study was: To date, what evidence exists on healthcare providers’ and managers’ perceptions of the quality of care in the free maternal healthcare era in SSA? Table 1 shows the framework (population, content, and context (PCC)) used to ascertain the suitability of the review question.

**Table 1:** PCC framework for defining the eligibility of the studies for the primary research question

P-Population	Frontline managers: All categories of health mangers such as Administrators, Medical Directors/Superintendents, Nurse Managers, and others Health providers: Doctors, Nurses, Pharmacies, Biomedical Scientist, and others.
C-Concept	Free maternal healthcare financing: refers to any health policy that allows women to access maternal health services during pregnancy, delivery, and post-natal period at no cost to them or their family members [17].
C-Context	Quality of care: It is how best the frontline managers and providers rank their expectations to the required standard regarding the quality of care rendered to their clients.

### Literature search

We searched five electronic databases (CINAHL through EBSCOhost, PubMed, Web of Science, ScienceDirect, and Google Scholar) with no date limitation to May 2019 for relevant articles (Supplementary file 1). We used a combination of the following keywords: “free maternal healthcare financing”, “healthcare financing”, “maternal healthcare”, “delivery”, “health service”, “managers”, “healthcare providers”, “quality of service delivery”. Boolean operators and medical subject headings were applicable were included in the search strategy. Limitations on language and study design were removed. We also searched the reference list of the included articles for eligible studies.

### Eligibility criteria

The inclusion criterion was that an article had to be written and published in English, involve at least one SSA country, include health providers/managers or both, and focus on a free maternal healthcare financing policy and quality of care. This review was limited to primary study designs (quantitative, qualitative, and mixed methods study). We excluded articles focused on clients’ perception of the quality of care.

### Study selection

The database search and the title screening were conducted by MAM using the eligibility criteria. Then, duplicates were deleted and the clean library was shared with the review team. Abstracts and full articles were screened independently by MAM and FID using tools pilot tested by the review team. The review team members discussed the discrepancies that arise out of the abstract screening between MAM and FID until a consensus was reached, while DK addressed the discrepancies at the full text phase.

### Charting the data

We extracted the following: author and publication year, country where the study was conducted, study design type, study setting, study population, and type of free maternal healthcare policy (fully or partially free). We also extracted the findings relevant to answer the review question using a deductive approach. To ensure the accuracy and trustworthiness of this study’s results, MAM and DK independently abstracted the data with BV acting as the arbiter.

### Collating and summarizing the results

Thematic analysis was conducted following the data extraction. The data were collated into themes and a summary of the study outcomes reported in narrative form.

## Results

**Fig. 1:**
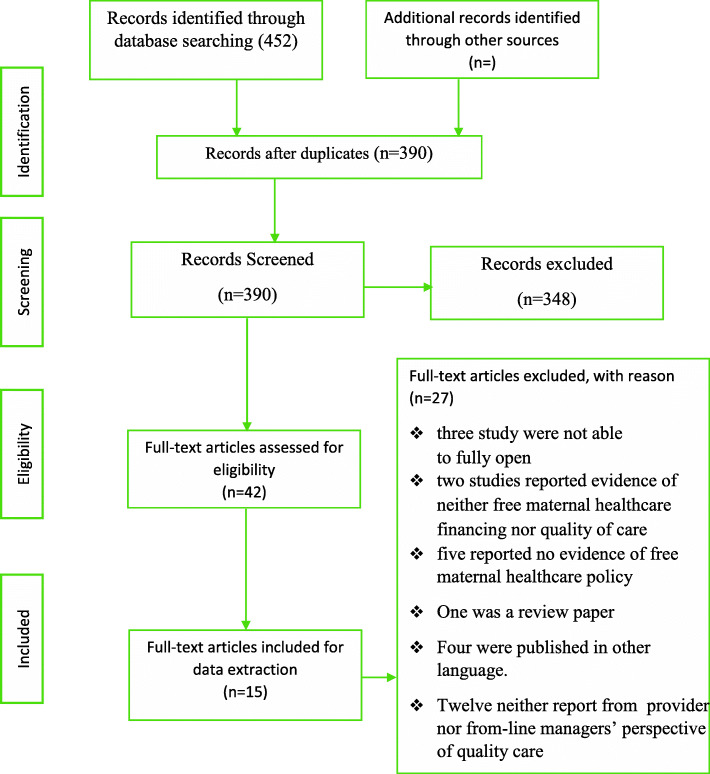
PRISMA 2009 Flow Diagram

Of the 452 eligible articles obtained from the database search, 62 duplicates were removed. Subsequently, 348 and 27 articles were removed from the abstract and full article screening stages respectively (Fig. 1). The reasons for exclusion following the full article screening were: inability to access the full text of three studies [18–20]; one was a protocol [21]; one was review paper [14]; fifteen articles did not report on either free or quality of maternal healthcare [5, 22–35];, and seven had no documentation on either front-line managers’ or providers’ perspective of free maternal healthcare [11, 36–41].

### Characteristics of the included studies

Among the 15 included articles that qualified for this study, the highest 26% (4) reported from Ghana, followed by 19% (3) and 13% (2) from Kenya and Nigeria respectively. The remainder (40%) of the 15 included articles were conducted in Burkina Faso, Burundi, Niger, Sierra Leone, Tanzania, and Uganda with 7% (1) each. Out of the 15 studies, 7 were conducted in health facility-based settings representing 53.3% [40, 42–47], 4 in community setting indicating 26.7% [48–51], whereas 2 (13.3%) were nationwide surveys [52, 53] and 1(6.7%) was a household survey [42]. Of the 15 include studies majority (40.0%) were mixed method studies [43, 46, 49, 51, 53, 54], qualitative studies (33.3%) [42, 45, 47, 48, 52], and cross-sectional studies (26.7%) [2, 40, 44]. The majority 9 (60.3%) were generally conducted among healthcare providers [2, 40, 43, 44, 47–49, 54], 1 (6.7%) among health managers [46], 5 (33%) involved both health providers and managers [42, 45, 51, 52, 55].

### Study findings

Out of the 15 included studies, only one reported that providers were satisfied with the quality of maternal healthcare [55]. The remaining 14 studies reported different challenges with the free maternal healthcare policy implementation which consequently resulted in a poor quality of maternal care [2, 40, 42–49, 51, 53, 54] (Table 2).

**Table 2:** Study findings

Author & date	Study pop.	Type of maternal healthcare financing policy	Significant study findings	Perception of quality of care
Dalinjong et al. 2018 [51]	Providers and managers	Free maternal healthcare	No strengthening of health system before implementing the free maternal health policy, facilities at the peripherals were not adequately resourced and lack of essential inputs.	Poor
Ganle et al. 2014 [48]	Providers	Free maternal healthcare	Limited and inequitable distribution of skilled maternal services, increased workload and difficulties in arranging the proper transport for referral cases	Poor
Nabyonga-Orem et al. 2008 [49]	Providers	Free maternal healthcare	Irregular drug and injectable supply, no fuel to facilitate providers movement and no allowances for staff	Poor
Nimpagaritse and Bertone 2011 [46]	Managers	Free maternal healthcare	Increase utilization of service delivery, high workload of providers and delay of reimbursement. No clear definition of the policy	Poor
Okonofua et al. 2011 [53]	Providers	Partial free maternal healthcare	Inadequate and improper allocation of funding	Poor
Pyone et al. 2017 [45]	Providers and managers	Free maternal healthcare	Weak enforcement mechanism, and lack of clarity of policy, delay in reimbursement and increased workload of providers with no allowances	Poor
Wamalwa 2015 [44]	Providers	Free maternal healthcare	No additional staff with overwhelming workload with no allowance, shortage of logistics, and delay in reimbursement	Poor
Dalinjong and Laar 2013 [2]	Providers	Free maternal healthcare	High utilization of service delivery of the insured. Delay in reimbursement, long working hours for providers without any motivation	Poor
Korom et al. 2017 [40]	Providers	Free maternal healthcare	Inadequate beds, and drugs supplies, no delivery rooms, no portable water.	Poor
Ogbuabor and Onwujekwe 2018 [52]	Providers and managers	Free maternal healthcare	No Health Facility Committee (HFC) participation, low awareness of level of funding, and weak legal framework	Poor
Belaid and Ridde 2015 [55]	Providers and managers	Partially free obstetric care	Staff strengthening and providers integration into the community	Good
Ridde and Diarra 2009 [43]	Providers	Free maternal healthcare	Health providers partially` object to the abolition of user-fee, perception of unsustainability of policy. Poor coordination of the availability of free maternal service at different levels in the health pyramid	Poor
Witter et al. 2013 [47]	Providers	Free maternal healthcare	Tariffs inadequacy from health insurance, location of facilities skewed in favour of those within urban centers, no financial support for the programme and increased workload of providers	Poor
Kuwawenaruwa et al. 2019 [54]	Providers	Free maternal healthcare	Overcrowding leading to unfilled forms, no allowance for extra duties. Limited training for providers, delay of reimbursement	Poor
Lang’at and Mwanri 2015 [42]	Providers and managers	Free maternal healthcare	Delays in reimbursement by the government to the facility, stock out of essential drugs, increase workload amidst staff shortage and no motivation	Poor

### Providers’ perspectives of the quality of maternal care

One of the most challenging problems faced by providers was a delay in reimbursement and/inadequacy of funds for free maternal healthcare policy. Two (2) studies, one in Kenya: Wamalwa, 2015, and another in Nigeria: Okonofua et al., 2011, stated the inadequacy of provision of funds for providers to render good quality care to women [44, 53] Moreover, Kuwawenaruwa et al., 2019 study in Tanzania also reported poor quality of care due to delays in reimbursement [54]. Nabyonga-Orem et al., 2008 wrote that in Uganda, an inadequate supply of essential drugs was persistent relative to Koroma et al., 2017 study from Sierra Leone. According to Wamalwa 2015, Nabyonga-Orem et al., 2008, and Kuwawenaruwa et al., 2019 studies in Kenya, Uganda, and Tanzania respectively, health providers never enjoyed any form of motivation [44, 49, 54]. In Ghana, Witter et al. 2013, and Ganle et al. 2014 reported that workload increased tremendously as a result of high utilization and shortage of staff in most facilities [47, 48]. Ganle et al., 2014, wrote that there was a shortage of staff in most facilities [48]. Due to the high utilization of care by pregnant women, the workload of providers increased greatly which affected the quality of care negatively [46]. In Kenya, Wamalma 2015, reported a heavy workload of providers as a result of high uptake of service with no additional staff to cope with the increasing rate of service utilization [44].

### Health managers’ perspectives on the quality of maternal care

Nimpagaritse and Bertone study in Burundi indicated that as a result of the complexity involved in recouping funds, out-of-pocket payment existed at the expense of the poor pregnant women [46]. This study further added that the quality of care was low because there was no clear definition of free maternal care policy [46]. Again, the study was emphatic on administrative staff shortage, and providers combining some administrative work which consequently render providers’ duties more burdensome [46].

### Quality of maternal care from both healthcare managers and providers viewpoint

Belaid and Ridde study in Burkina Faso showed that healthcare providers and managers had a good perception of the quality of maternal healthcare [55]. Factors contributing to the good quality of care and increasing facility-based deliveries were attributed to leadership, strengthening relationships of trust with communities, users’ positive perceptions of quality of care, and the introduction of female professional staff [55]. Aside from this commendation, all other included studies involving the managers, and providers or both ranked the quality of service as low [2, 40, 42, 44–46, 48, 49, 51–56]. Pyone et al., Lang’at and Mwanri, and Dalinjong and Laar in their respective studies reported delay of reimbursement with the advent of free maternal healthcare implementation affecting service delivery [2, 42, 45]. Another problem affecting the quality of care was the provision of essential drugs. In Ghana, Dalinjong et al. observed a stockout of essential drugs due to the introduction of free maternal healthcare [51]. Similarly, Lang’at and Mwanri reported an irregular supply of essential drugs in Kenya [42]. Moreover, few of the included studies reported that there was no staff motivation/allowances despite the increased workload as a result of the high utilization of care by mothers [42, 44, 45, 49, 54]. Dalinjong et al. also observed there was no motivation for providers and managers in Ghana.

In Nigeria, Ogboubor and Onwujekwe observed that Health Facility Committees were not involved in the fund generation, management, and tracking of expenditure [52]. Dailinjong and Laar observed that the increased workload of providers caused much negative influence on the insured pregnant women [2]. Due to stress and fatigue on the side of providers, pregnant women suffered verbal abuse [2]. Notwithstanding, pregnant women prefer the older midwives to the newly trained which consequently resulted in staff overstretched [2]. Lang’at et al., remarked that out of the overwhelmed workload, complications sometimes occur despite early reports by mothers [42]. Pyone et al. indicated that resource management was burdensome due to the busy schedules of health providers [45].

## Discussion

Our study was conducted to describe existing literature on free maternal healthcare and the quality of care based on healthcare managers’ and providers’ perspectives in SSA. We found 15 studies from 9 out of the 46 countries in SSA. The study result revealed that the majority (93.3%) of the included studies indicate the quality of maternal healthcare was poor under the free financing policy era [2, 40, 42–49, 51–54]. We found limited studies reporting on managers’ and providers’ perceptions of the quality of maternal care offered to mothers under the free financing policy. Healthcare managers and providers are key stakeholders in the healthcare industry and their perspectives on any healthcare policy are vital to ensure the provision of quality care. Therefore, primary research involving them focusing on the quality of maternal care is needed. Moreover, to achieve the SDG 3.1 target, there is the need to dive into the quality of care as expected by healthcare managers and providers since they have an overwhelming influence on policy decision-making and improvement in maternal health. Among the 46 countries in SSA, evidence was found in the following countries: Ghana, Nigeria, Niger, Burkina Faso, Burundi, Sierra Leone, Kenya, Tanzania, and Uganda. The literature suggests that free maternal health services exist in 19 SSA countries in different forms [56–59]. Based on this study’s inclusion criteria, we found evidence from only seven countries representing about 37% of those 19 countries. Managers and providers perceived that delay in reimbursement, inequitable distribution of health facilities, unclear definition of free maternal healthcare policy, inadequate provision of essential drugs and supplies, and limited training of providers ranked the quality of care as poor. Notwithstanding, the governments in SSA’s ability to solve these major challenges could improve maternal healthcare quality.

### Implication for practice

This study’s findings suggest that the majority of healthcare managers’ and providers’ perspectives of quality of care in the included studies were not up to standard. The increased workload on providers might have contributed to long waiting times for clients and providers’ ill-attitudes due to stress. This problem may cause a decrease in utilization and loss of trust for the providers. Moreover, delays in reimbursement and lack of essential drugs would have several implications such as service ineffectiveness, OOP payment, and low quality of care. The persistence of such problems can also lead to decrease service utilisation. Perhaps, a lack of an explicit policy definition could have accounted for the lack of provision of other services that could enhance service quality. Therefore, this study recommends practical solutions to address the challenges facing the implementation of a free maternal healthcare policy towards achieving SDG 3.1.

### Implication for research

This study suggests limited primary research evaluating health providers’ and managers’ perceptions of the free maternal healthcare policy and the quality of maternal care in SSA. The sustainability of a free maternal healthcare policy is key to removing financial barriers to maternal health services. We, therefore, recommend more primary research involving all stakeholders that aim at understanding their perceptions of the free maternal healthcare policy and their impact on the quality of care in those SSA countries where the policy exists. This study also recommends more research to understand the implementation challenges of free maternal healthcare as well as recommend evidence-based solutions to address them in those SSA countries the policy exists.

### Strengths and limitations

A scoping review permits the inclusion of different study designs. Our chosen study method allowed us to systematically searched for and selected relevant literature to describe the evidence on the quality of care in the free maternal healthcare era in SSA focusing on the perspectives of healthcare providers and managers. This study design further allowed to establish literature gaps useful to inform future research. To the best of our knowledge, this study is the first of its kind to scope literature focusing on healthcare providers and managers and their perception of the quality of maternal healthcare in the free financing era. Notwithstanding these strengths, this study’s limitations are many. These limitations are published elsewhere [15]. Also, we possibly did not capture some relevant articles since we search fewer databases. Moreover, the study was limited to healthcare managers’ and providers’ perspectives of free maternal healthcare and quality of care even though there are other stakeholders where important information could have been retrieved. The use of “free maternal healthcare” as a keyword potentially excluded some articles. Despite all these limitations, we the evidence provided by this review is useful to guide future research.

## Conclusion

This study established evidence of existing literature on the quality of care based on Healthcare providers’ and managers’ perspectives though very limited. This study indicates healthcare providers and managers perceive the quality of maternal healthcare under the free financing policy is poor. They expected early reimbursement of funds, a clear definition of policy, equitable distribution of health facilities and health workforce, availability of essential drugs and logistics, and risk allowances in order to rank service quality as good. More research is needed to better understand the impact of their perceived poor quality of care on maternal health outcomes. Nonetheless, the free maternal care policy is very much needed towards achieving universal health, and all efforts to sustain and improve the quality of care under it, must be encouraged.

## Supplementary Information

**Additional file 1: Supplementary file 1**. Electronic databases search and title screening results

## Data Availability

All materials and data used for this study have been provided in the reference list.

## Abbreviations

LMIC: Low-and-Middle Income Countries
HFC: Health Facility Committee
MDG: Millennium Development Goal
MMAT: Mixed Method Quality Appraisal Tool
MMR: Maternal Mortality Rate
OOP: Out-of-Pocket
PCC: Population, Content and Context
PRISMA-ScR: Preferred Reporting Items for Systematic Reviews and Meta-Analyses modified for Scoping Reviews
SDGs: Sustainable Development Goals
SSA: Sub-Saharan African
TBAs: Traditional Birth Attendants
WHO: World Health Organization
