# Supplementary material for: Quality of care in the free maternal healthcare era in sub-Saharan Africa: a scoping review of providers’ and managers’ perceptions
Source: BMC Pregnancy Childbirth. 2021 Mar 19;21:220. doi: 10.1186/s12884-021-03701-z (PMC7977170; doi:10.1186/s12884-021-03701-z)
Supplement: Supplementary file 1 — Additional file 1: Supplementary file 1. Electronic databases search and title screening results [file 12884_2021_3701_MOESM1_ESM.docx]

**Supplementary file 1:** Electronic databases search and title screening results

| **Date** | **Databases** | **Keywords** | **Search results** | **Eligible studies** |
| --- | --- | --- | --- | --- |
| 12/05/2019 | PubMed | ("healthcare financing"[MeSH Terms] OR ("healthcare"[All Fields] AND "financing"[All Fields]) OR "healthcare financing"[All Fields]) AND ("quality of health care"[MeSH Terms] OR ("quality"[All Fields] AND "health"[All Fields] AND "care"[All Fields]) OR "quality of health care"[All Fields] OR ("quality"[All Fields] AND "care"[All Fields]) OR "quality of care"[All Fields]) | 2,010 | 71 |
| 12/05/2019 | PubMed | (free[All Fields] AND ("mothers"[MeSH Terms] OR "mothers"[All Fields] OR "maternal"[All Fields]) AND ("delivery of health care"[MeSH Terms] OR ("delivery"[All Fields] AND "health"[All Fields] AND "care"[All Fields]) OR "delivery of health care"[All Fields] OR "healthcare"[All Fields])) AND ("quality of health care"[MeSH Terms] OR ("quality"[All Fields] AND "health"[All Fields] AND "care"[All Fields]) OR "quality of health care"[All Fields] OR ("quality"[All Fields] AND "care"[All Fields]) OR "quality of care"[All Fields]) | 850 | 42 |
| 14/05/2019 | Science Direct | free maternal health care and quality of care | 24,831 | 63 |
| 16/05/2019 | Google Scholar | women perception OR health provider perception OR health manger perception AND free maternal healthcare | 18,100 | 98 |
| 17/05/2019 | Google Scholar | free maternal healthcare financing AND perceive quality of care | 23,000 | 96 |
| 18/05/2019 | Web of Science | Free maternal health service AND Pregnant women OR expectant mothers AND quality of care | 1303 | 30 |
| 19/05/2019 | CINAHL | women perception OR perception AND free maternal healthcare | 361 | 52 |
| **Total** |  |  | **70,455** | **452** |
